# Characterization and transcriptomic analysis of a novel yellow-green leaf wucai (*Brassica campestris* L.) germplasm

**DOI:** 10.1186/s12864-021-07573-7

**Published:** 2021-04-12

**Authors:** Libing Nie, Yushan Zheng, Liting Zhang, Ying Wu, Shidong Zhu, Jinfeng Hou, Guohu Chen, Xiaoyan Tang, Chenggang Wang, Lingyun Yuan

**Affiliations:** 1 College of Horticulture, Vegetable Genetics and Breeding Laboratory, Anhui Agricultural University, 130 West Changjiang Road, Hefei, 230036 Anhui China; 2 Provincial Engineering Laboratory for Horticultural Crop Breeding of Anhui, 130 West of Changjiang Road, Hefei, 230036 Anhui China; 3 Wanjiang Vegetable Industrial Technology Institute, Maanshan, 238200 Anhui China

**Keywords:** Transcriptome, *Brassica campestris* L. ssp. *chinensis* var. *rosularis*, Chlorophyll biosynthesis, Photosynthesis, Yellow-green leaf mutant

## Abstract

**Background:**

Leaf color mutants are the ideal materials to explore the pathways of chlorophyll (Chl) metabolism, chloroplast development, and photosynthesis system. In this study, a spontaneous yellow-green leaf wucai (*Brassica campestris* L.) mutant “WY16–13” was identified, which exhibited yellow-green leaf color during its entire growth period. However, current understanding of the molecular mechanism underlying Chl metabolism and chloroplast development of “WY16–13” is limited.

**Results:**

Total Chl and carotenoid content in WY16–13 was reduced by 60.92 and 58.82%, respectively, as compared with its wild type parental line W16–13. Electron microscopic investigation revealed fewer chloroplasts per cell and looser stroma lamellae in WY16–13 than in W16–13. A comparative transcriptome profiling was performed using leaves from the yellow-green leaf type (WY16–13) and normal green-leaf type (W16–13). A total of 54.12 million (M) (WY16–13) and 56.17 M (W16–13) reads were generated. A total of 40,578 genes were identified from the mapped libraries. We identified 3882 differentially expressed genes (DEGs) in WY16–13 compared with W16–13 (i.e., 1603 upregulated genes and 2279 downregulated genes). According to the Gene Ontology (GO) term and Kyoto Encyclopedia of Genes and Genomes (KEGG) pathway analyses, these DEGs are involved in porphyrin and Chl metabolism [i.e., chlorophyllase (*CLH*), heme oxygenase (*HO*), chlorophyll (ide) b reductase (*NYC*), and protochlorophyllide oxidoreductase (*POR*) genes], carbohydrate metabolism, photosynthesis, and carbon fixation in photosynthetic organisms. Moreover, deficiency in Chl biosynthetic intermediates in WY16–13 revealed that the formation of the yellow-green phenotype was related to the disorder of heme metabolism.

**Conclusions:**

Our results provide valuable insights into Chl deficiency in the yellow-green leaf mutant and a bioinformatics resource for further functional identification of key allelic genes responsible for differences in Chl content.

**Supplementary Information:**

The online version contains supplementary material available at 10.1186/s12864-021-07573-7.

## Background

Leaves are crucial organs that produce photosynthates for plant development and growth. Leaf color is mainly determined by pigment types and their relative concentrations. Chlorophyll (Chl) is the main pigment in leaves and are the primary photoreceptor pigments that capture light energy and drive electron transfer in the reaction center to form chemical energy and synthesize carbohydrates [1, 2]. In *Arabidopsis thaliana*, 27 genes involved in Chl metabolisms were identified, starting from glutamyl-tRNA to Chl a and Chl b [2].

Recently, mutants with disrupted Chl biosynthesis and degradation have been used to characterize steps associated with Chl metabolism in yellow-leaf plants such as rice [3–5], *Arabidopsis thaliana* [6], and pak-choi [7]. Numerous studies on other crops have been conducted to elucidate the molecular mechanisms of leaf color mutants, particularly leaf yellowing. Yellow-green leaf mutants usually exhibit Chl deficiencies, shorter plant height, and retarded growth, resulting in decreased yield or even in plant death [8–10]. For instance, a yellow-green leaf phenotype is attributed to the disruption of the Chl synthase-encoding gene *YGL1* or impaired chlorophyllide esterification, which results in lower Chl content and delayed chloroplast development [11, 12]. Virus-induced gene silencing of magnesium chelatase subunit D (*CHLD*) and I (*CHLI*) results in yellow phenotypes in peas with reduced Chl content and altered chloroplast function with abnormal chloroplast structure [13]. In rice, the pale-green leaf mutant *pgl10* that is deficient in protochlorophyllide oxidoreductase (PORB) results in lower photosynthetic pigment content and grana lamellae in thylakoid compared to the wild-type [14]. Moreover, both external and internal factors could also influence Chl content such as light, salt, and osmotic stress due to alterations in gene expression or post-translational modification of proteins involved in Chl metabolism [15–17]. For example, a rice mutant deficient in fructose-1,6-bisphosphatase (FBFase) exhibits a yellow-green leaf phenotype and severe growth retardation [18]. In addition to alterations in Chl metabolism, the disruption of chloroplast function could also negatively affect Chl content and stability [15, 17]. The chloroplast plays a profound role in the biosynthesis of hormones, carbohydrates, and amino acids, as well as in energy metabolism. Peroxisomes, mitochondria, and chloroplast work cooperatively in plant energy metabolism pathways. Snowy cotyledon 3 (SCO3) localizes to peroxisomes, and its mutation in *Arabidopsis* leads to loss of Chl content and defective chloroplast function [19].

**Fig. 1:**
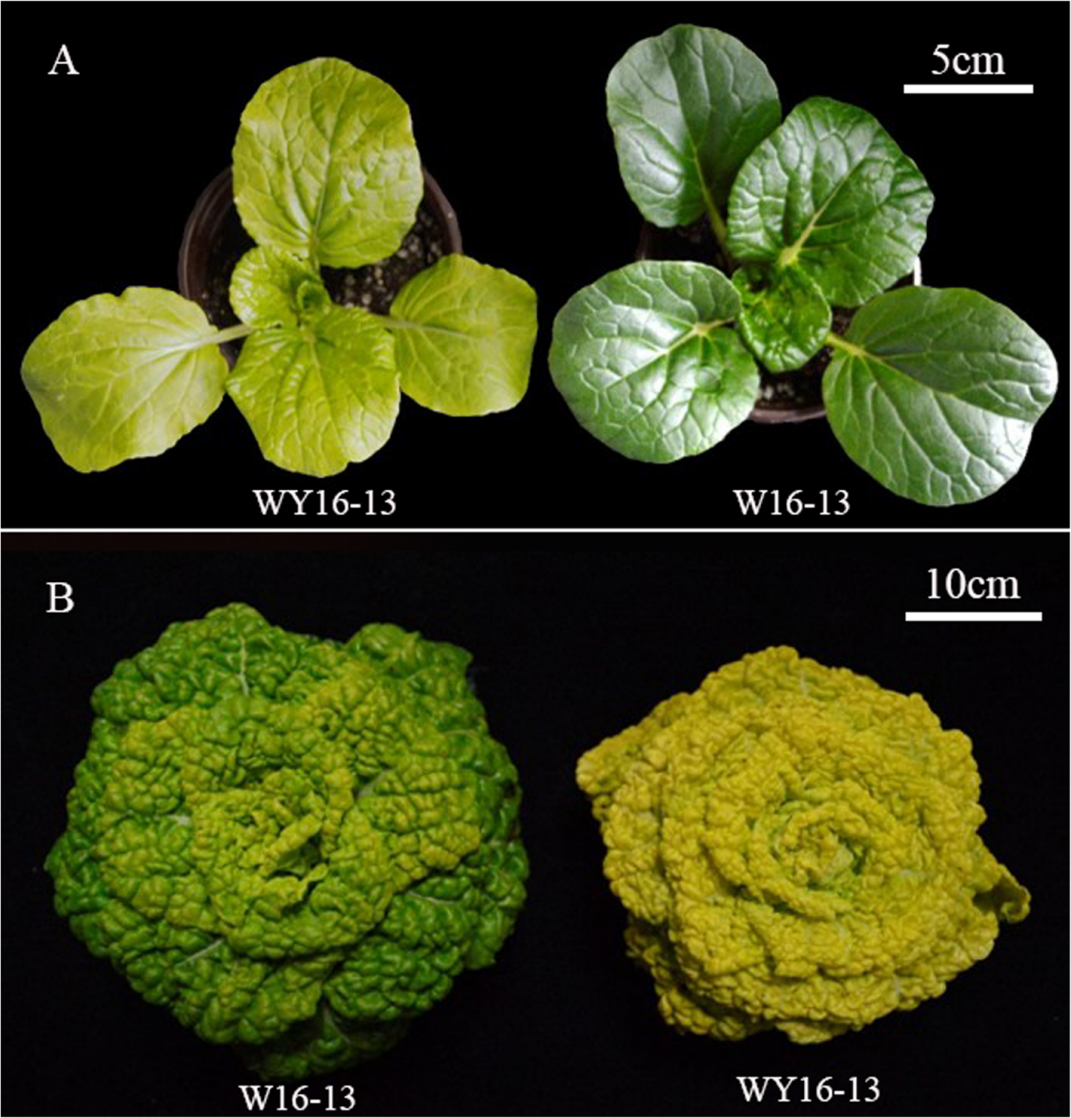
Phenotype characterization of yellow-green leaf mutant WY16–13 and wild-type W16–13; WY16–13 and W16–13 at (**a**) seedling stage, Scale bar: 5 cm; and (**b**) harvesting stage, Scale bar: 10 cm

Wucai (*Brassica campestris* L. ssp. *chinensis* var. *rosularis*) belongs to non-heading Chinese cabbage family and is broadly cultivated in the Yangtze-Huai River basin in winter because of its cold-tolerant and high-quality features [20, 21]. It has a variety of leaf colors and usual colors in the adult stage include dark green, green, outer dark green-inner green, outer green-inner light green, and outer light green-inner yellow. Currently, a novel wucai germplasm naturally occurs from a self-pollinated, stable, genetic high-generation inbred line “W16–13.” It possesses a stable yellow-green leaf phenotype in the offspring at whole developmental stages and has been identified as “WY16–13” (Fig. 1). Its hybrid F_1_ line, crossed with another green-leaf line, exhibits yellow-colored in inner leaves and green-colored in outer leaves and hybrid vigor. Therefore, “WY16–13” is an alternative and ideal yellow-green wucai germplasm to consider in future breeding programs and plantations. However, the molecular mechanism underlying its phenotype remains unclear.

In our study, the photosynthetic pigments, chloroplast ultrastructure, enzyme activity, and intermediate metabolites involved in Chl biosynthesis were assessed, and transcriptome level changes in WY16–13 and W16–13 were analyzed. Based on a combination of physiological and bioinformatics analyses, we identified DEGs related to Chl biosynthesis, and transcript levels of some key genes were evaluated to validate their involvement in leaf coloration. Our results illustrated the physiological and transcriptomic aspects of yellow-green leaf coloration in wucai and provide novel insights into the mechanism underlying Chl metabolism and chloroplast development.

## Results

### Phenotypic characterization of the WY16–13 mutant

**Fig. 2:**
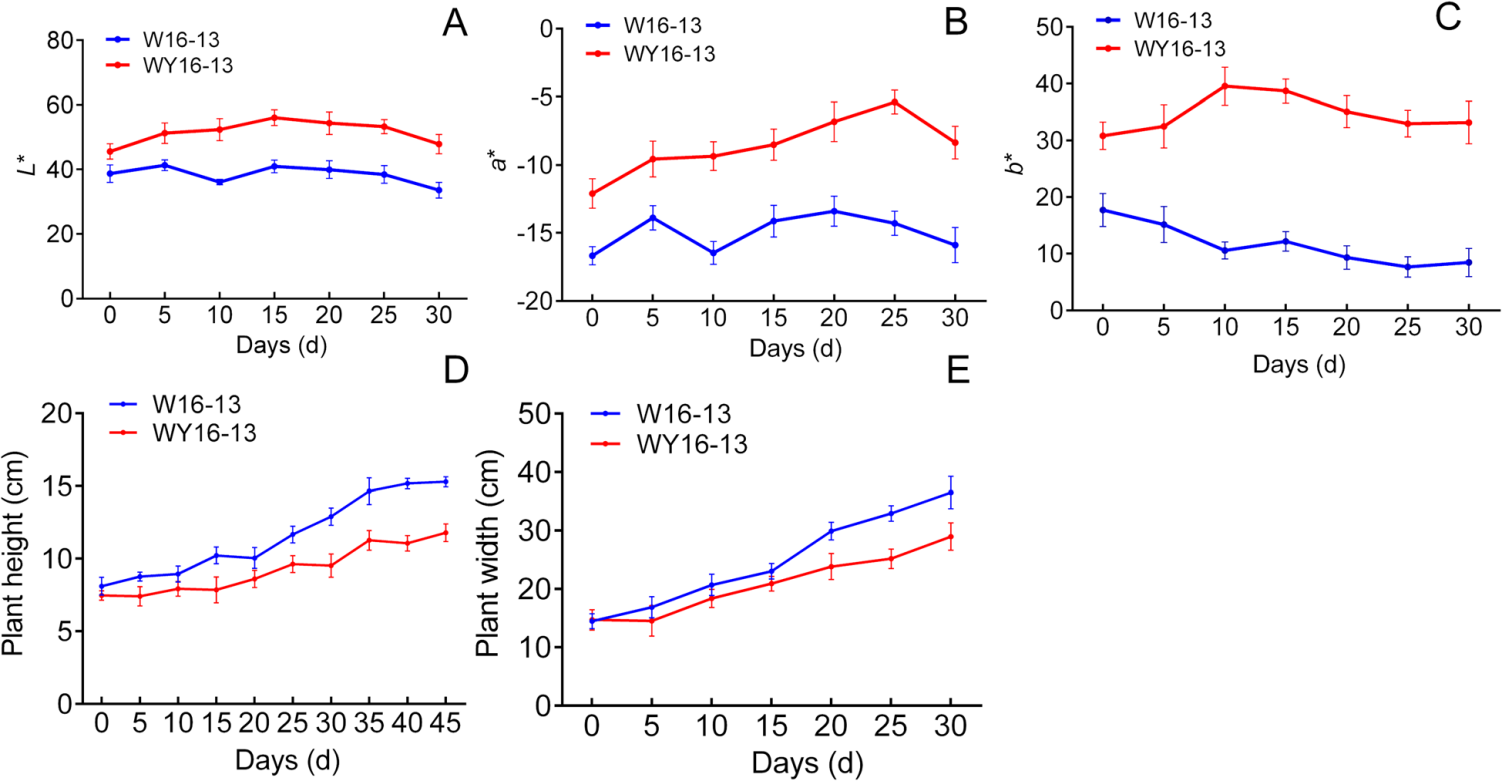
Comparison of L^*^ (**a**), a^*^ (**b**), b^*^ (**c**), plant height (**d**), and plant width (**e**) between WY16–13 and W16–13 at different days after planting. Error bars represent SD (±SD), and representative data from five independent experiments are shown

Compared with W16–13, the leaves of WY16–13 exhibited yellow color during the entire growth period (Fig. 1). The color parameters L^*^, a^*^, and b^*^, which represented brightness, greenness, and yellowness, respectively, were significantly different between W16–13 and WY16–13. Compared with W16–13, L^*^ and b^*^ of WY16–13 were markedly higher, whereas a^*^ was lower during the entire growth period (Fig. 2a–c). Additionally, measurement of the growth parameters showed that height and weight of WY16–13 were significantly lower than W16–13 throughout growth (Fig. 2d and e). Compared with W16–13, height of WY16–13 decreased by 23.09% at 35 d. Similarly, WY16–13 width decreased by 23.57% at 25 d.

**Fig. 3:**
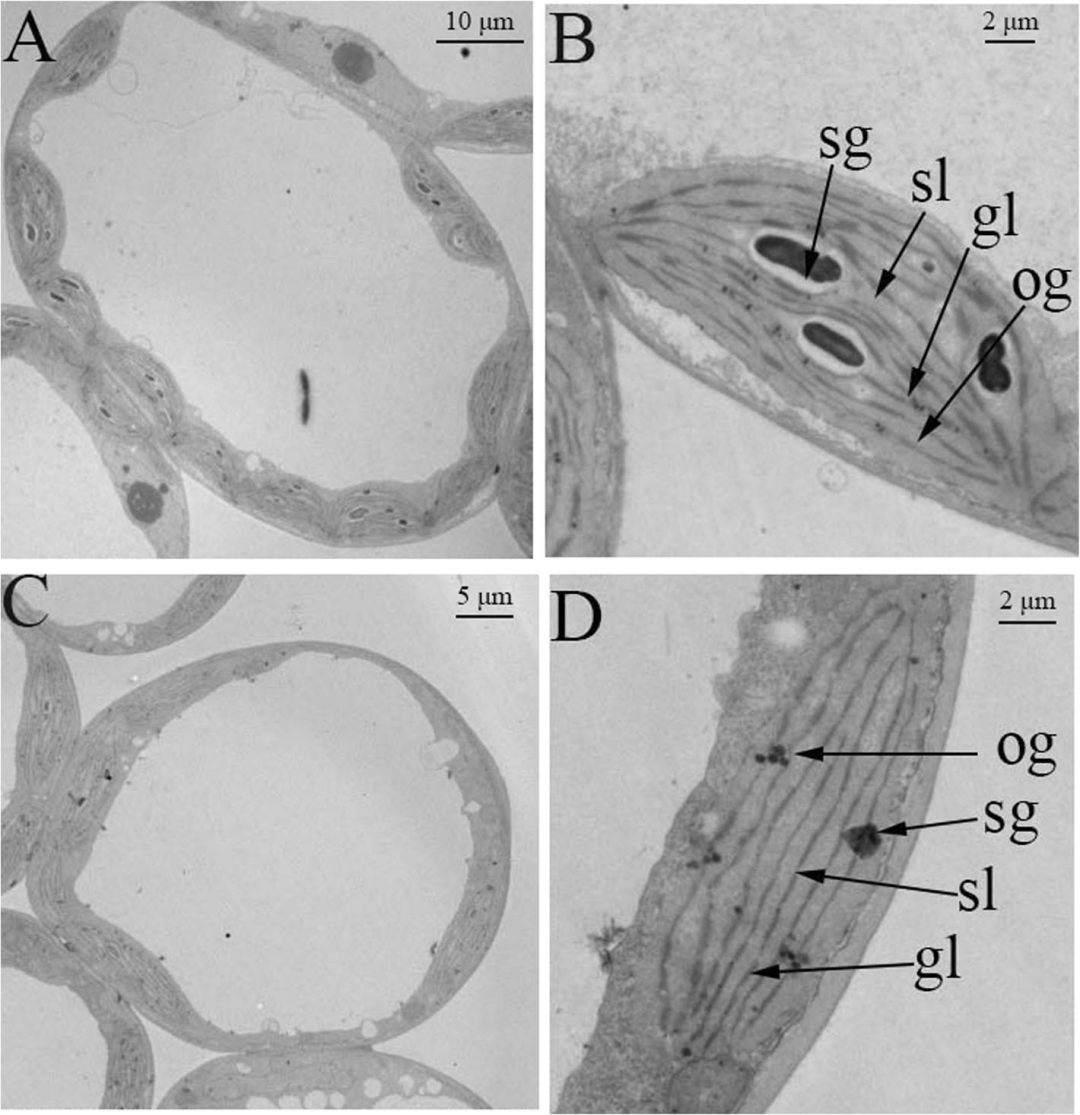
Transmission electron micrograph of chloroplasts from mutant (WY16–13) and wild-type (W16–13) wucai. Structure of mesophyll cells in W16–13 (**a**) and WY16–13 (**c**). Structure of chloroplasts in W16–13 (**b**) and WY16–13 (**d**). sg: starch grains, sl: stroma lamellae, gl: grana lamellae, og: osmiophilic granules. **a** Bar: 10 μm; **b** Bar: 2 μm; **c** Bar: 5 μm; **d** Bar: 2 μm

Assessment of chloroplast ultrastructure of the WY16–13 and W16–13 leaves was performed by the transmission electron microscopy (Fig. 3). Compared with the W16–13, WY16–13 exhibited fewer chloroplasts per cell and looser stroma lamellae. In addition, we observed a decrease in the number of starch granules, an increase in the number of osmiophilic granules, and the lack of grana membranes in the chloroplasts of WY16–13 compared with the W16–13. These results indicated that the development of chloroplast was defective in WY16–13, which in turn disrupts plant photosynthesis.

### Chl metabolism analysis

In the present study, the Chl and Carotenoid (Car) contents in W16–13 and WY16–13 leaves were measured (Table 1). The Chl and Car contents of the green leaves were significantly higher than those of the yellow leaves. Chl a, Chl b, and Car contents in WY16–13 significantly decreased by about 59.66, 64.81, and 58.82% relative to the leaves of the W16–13, respectively. These results suggest that the yellow mutant phenotype is probably due to reduced contents of photosynthetic pigments.

**Table 1:** Contents and relative ratio of photosynthetic pigments of leaves in W16–13 and WY16–13

Material	Total Chl content	Chl a content	Chl b content	Car content	Chl a/Chl b	Chl/Car
W16–13	1.74 ± 0.12^**^	1.19 ± 0.06^**^	0.54 ± 0.06^**^	0.17 ± 0.01^**^	2.22 ± 0.16	9.94 ± 0.72
WY16–13	0.68 ± 0.01	0.48 ± 0.01	0.19 ± 0.01	0.07 ± 0.01	2.47 ± 0.09	9.05 ± 1.19

Error bars indicate means ± SD based on three independent experiments. Significant differences were determined using the Student’s *t* test in WY16–13 compared with W16–13 (**p < 0.01)

**Fig. 4:**
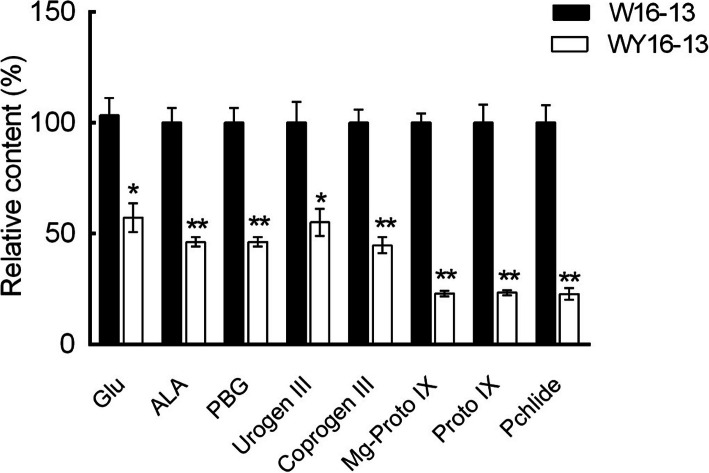
Comparison of the relative contents of chlorophyll precursors. Three individuals were measured for each chlorophyll precursor. Error bars indicate means ± SD based on three independent experiments. Significant differences were determined using the Student’s *t* test in WY16–13 compared with W16–13 (^*^ *p* < 0.05, ^**^ *p* < 0.01). Glu, Glutamate; ALA, 5-aminolevulinic acid; PBG, porphobilinogen; Urogen III, uroporphyrinogen III; Coprogen III, coproporphyrinogen III; Proto IX, protoporphyrin IX; Mg-Proto IX, Mg-protoporphyrin IX; Pchlide, protochlorophyllide

Furthermore, to investigate which biochemical step was disrupted and resulted in the yellow-leaf color phenotype, we assessed the levels of Chl biosynthesis intermediates in the W16–13 and WY16–13 leaves. Eight intermediate products that were related to Chl biosynthesis metabolic process were compared (Fig. 4). The results show that the Glutamate (Glu), 5-aminolevulinic acid (ALA), porphobilinogen (PBG), uroporphyrinogen III (Urogen III), coproporphyrinogen III (Coprogen III), Mg-protoporphyrin IX (Mg-Proto IX), protoporphyrin IX (Proto IX), and protochlorophyllide (Pchlide) contents of the leaves of WY16–13 plants were significantly lower than those of W16–13 plants. The levels of ALA and Proto IX in WY16–13 were only 46.24 and 23.27% of the W16–13, respectively.

### Sequencing and identification of expressed genes

To explore molecular mechanism of the yellow-leaf phenotype of WY16–13, cDNA libraries of W16–13 and WY16–13 were constructed and based on three biological replicates (Table 2). The total number of paired-end reads in the three biological replicates of the W16–13 was 56,516,916, 55,940,140, and 52,046,486, respectively, whereas that in WY16–13 was 51,448,202, 52,876,246, and 54,528,540. A total of 56.17 M (W16–13) and 54.12 M (WY16–13) reads were generated. Moreover, the three samples of W16–13 and WY16–13 have strong correlation (Additional file 1: Figure S1 and Additional file 2: Figure S2). Clean reads were obtained by removing the adapters, low-quality, poly-N, and empty reads, and the yield was about 7 G, with a GC percentage of about 47%. The Q30 of the raw data ranged from 92.64 to 93.75%, which indicated a high read confidence level. More than 88% (88.56–89.39%) of the reads in the two transcriptome libraries mapped to the *B. rapa* reference genome. A total of 40,578 genes were identified from the mapped libraries.

**Table 2:** The quality of the transcriptome of WY16–13 and W16–13 leaves

Sample	raw_reads	clean_reads	clean_bases	Q30	GC	Total number of reads	Total number of mapped reads
W16_13a	57.87 M	56.52 M	7.47 G	93.37%	48.47%	56,516,916	50,520,317 (89.39%)
W16_13b	57.33 M	55.94 M	7.38 G	93.65%	48.27%	55,940,140	49,750,259 (88.93%)
W16_13c	53.32 M	52.05 M	6.93 G	93.37%	48.39%	52,046,486	46,372,520 (89.10%)
WY16_13a	52.65 M	51.45 M	6.88 G	93.75%	47.92%	51,448,202	45,657,240 (88.74%)
WY16_13b	53.97 M	52.88 M	7.09 G	93.20%	47.82%	52,876,246	46,827,653 (88.56%)
WY16_13c	55.74 M	54.53 M	7.26 G	92.64%	48.04%	54,528,540	48,650,218 (89.22%)

### DEG identification

**Fig. 5:**
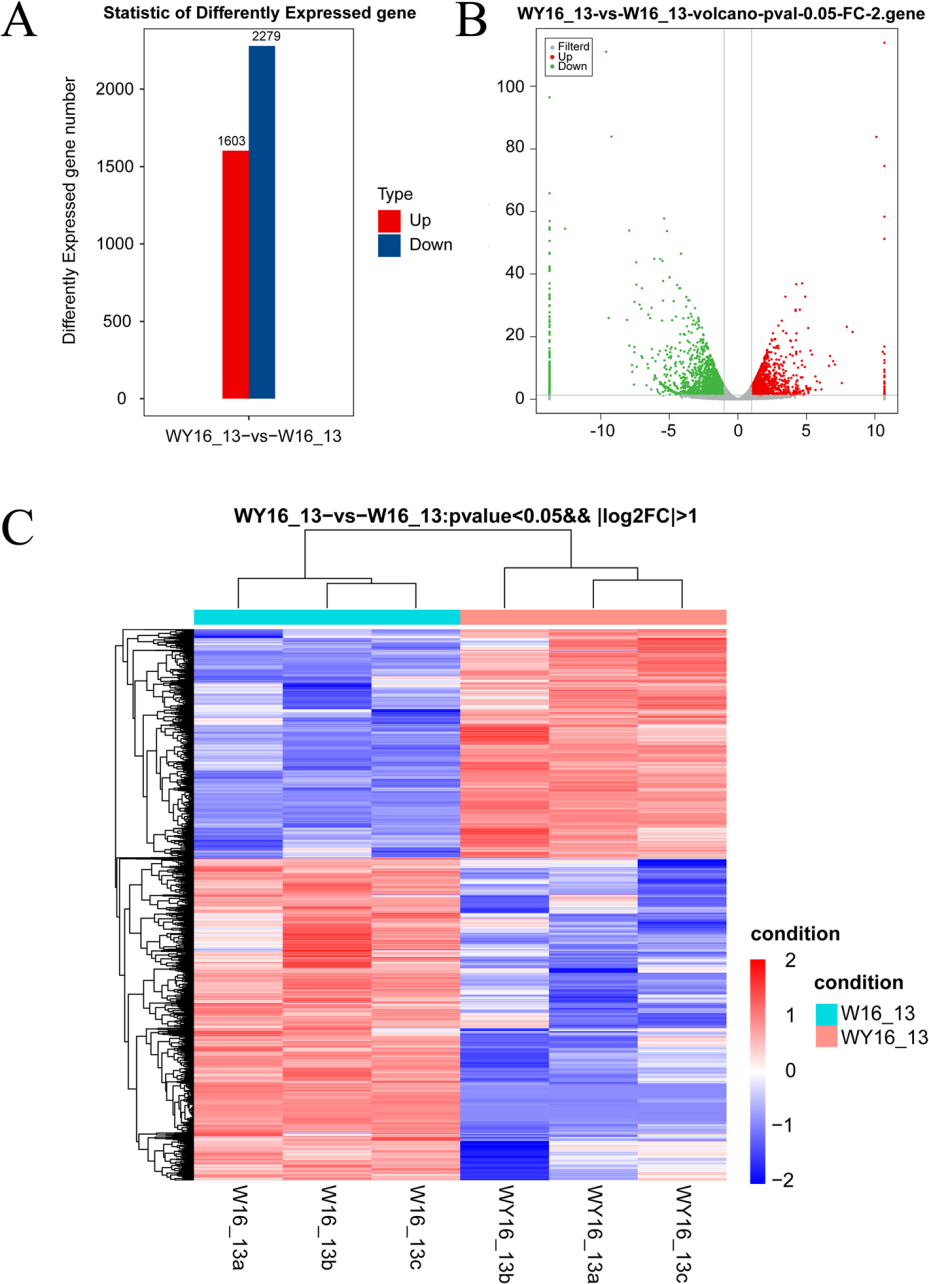
Transcriptome analysis of DEGs in WY16–13 and W16–13. Comparison of DEGs in WY16–13 and W16–13 (**a**). Volcano plot showing the DEGs between two different libraries (**b**). The threshold *q* < 0.05 was used to determine the significance of DEGs. Red and green dots represent up- and downregulated genes, respectively, and gray dots indicate transcripts that did not significantly change in the WY16–13 library compared to W16–13. Hierarchical clustering of all of the DEGs was based on the log_10_RPKM values (**c**). The color spectrum from blue to red represents gene expression intensity, ranging from low to high, respectively

Comparison of the yellow-green (WY16–13) leaves to the green (W16–13) leaves identified a total of 3882 DEGs, which included 1603 upregulated and 2279 downregulated genes (Fig. 5a and b). Hierarchical clustering of the DEGs was conducted to assess gene expression patterns and was calculated using the log_10_ RPKMs of the WY16–13 and W16–13 (Fig. 5c).

### GO and KEGG analyses of DEGs

**Fig. 6:**
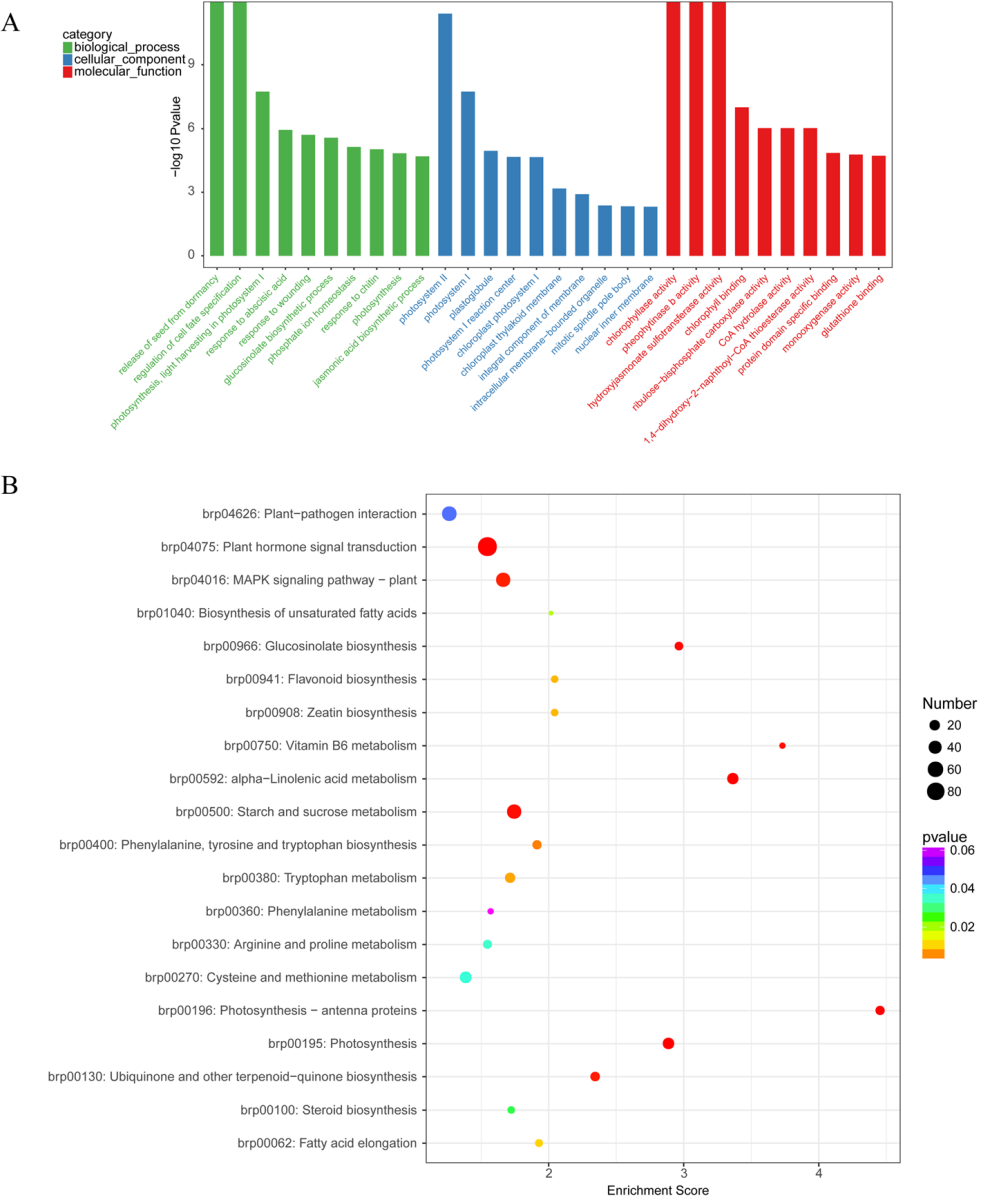
GO and KEGG pathway enrichment analyses of DEGs between WY16–13 and W16–13. GO enrichment analysis with the 30 most enriched GO terms in the three categories shown (**a**). KEGG enrichment analysis with the 20 most enriched KEGG terms shown (**b**). High and low *p*-values are represented by blue and red, respectively

To explore the DEGs involved in yellow-green coloration, GO assignments were applied to classify the functions of the DEGs. A total of 3882 DEGs were divided into biological processes, cellular components, and molecular functions. Some DEGs were annotated with more than one GO term (Fig. 6a). In the biological process category, many genes belong to “release of seed from dormancy (GO:0048838),” “regulation of cell fate specification (GO:0042659),” “photosynthesis, light harvesting in photosystem I (GO:0009768),” and “response to abscisic acid (GO:0009737).” Some genes related to cellular component progress were involved in “photosystem II (GO:0009523),” “photosystem I (GO:0009522),” and “plastoglobuli (GO:0010287).” The molecular function group mainly included “chlorophyllase activity (GO:0047746),” “phephytinase b activity (GO:0102293),” “hydroxyjasmonate sulfotransferase activity (GO:0080131),” and “chlorophyll binding (GO:0016168).”

KEGG pathway analysis was conducted to categorize gene functions with an emphasis on biochemical pathways that were active in yellow-green and green leaves. A total of 1001 DEGs were annotated and assigned to processes such as cellular processes, environmental information processing, genetic information processing, metabolism, and biological systems (Additional file 1: Figure S1). Most of the DEGs enriched the functional subcategory of metabolism (Fig. 6b). The most enriched pathway was “carbohydrate metabolism,” with 127 associated DEGs, followed by “amino acid metabolism” (94 DEGs) and “lipid metabolism” (84 DEGs). Many genes (111 DEGs) were belonged to the signal transduction group, which belongs to “environmental information processing.” Moreover, 74 DEGs were assigned to “folding, sorting, and degradation” and 53 DEGs were assigned to “environmental adaptation.” These results indicate that yellow-green and green leaves mainly differed in terms of metabolic processing. In addition, we focused on the analysis of DEGs related to chlorophyll metabolism. The results showed that Nine DEGs related to porphyrin and chlorophyll pathway (path: brp00860) were differentially expressed.

### Identification of DEGs related to Chl metabolism

**Fig. 7:**
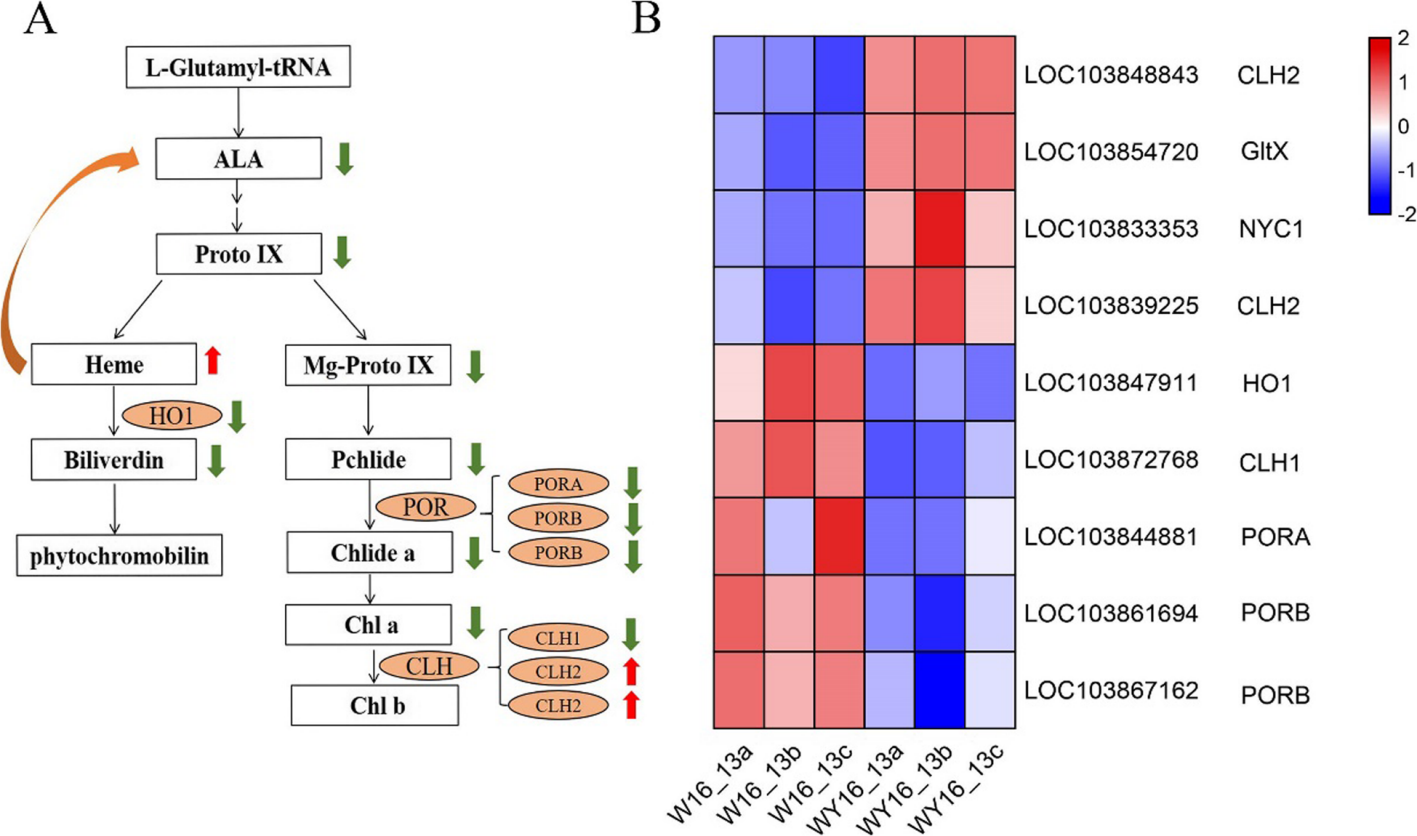
DEGs involved in the chlorophyll biosynthesis pathway. Chlorophyll biosynthesis pathway (**a**); upregulated genes are marked by red arrows and downregulated genes by green arrows. Expression profile clustering of the chlorophyll biosynthesis pathway (**b**). Expression ratios are based on log_2_ fragments per kilobase of transcript per million mapped reads (FPKM) values, where each vertical column represents a sample (W16_13a, W16_13b, and W16_13c; WY16_13a, WY16_13b, and WY16_13c), and each horizontal row represents a single gene. CLH, chlorophyllase; POR, protochlorophyllide oxidoreductase; HO, heme oxygenase; NYC, probable chlorophyll (ide) b reductase

Based on the above annotations, the DEGs in the porphyrin and the Chl pathway (path: brp00860) were compared in detail between the WY16–13 and W16–13 transcriptomes (Additional file 4: Table S1). The results demonstrated that five genes in the porphyrin and Chl metabolism pathway were downregulated (q < 0.05, fold change > 2), including two genes encoding PORB (*LOC103861694* and *LOC103867162*), one gene encoding PORA (*LOC103844881*), one gene encoding CLH1 (*LOC103872768*), and a gene encoding HO1 (*LOC103847911*) (Fig. 7b). Four DEGs were upregulated, encoding CLH2 (*LOC103848843* and *LOC103839225*), glutamate-tRNA ligase (*LOC103854720*), and NYC1 (*LOC103833353*) (Fig. 7b). These results indicated that green and yellow leaves largely differed in metabolic activities.

### Photosynthesis analysis

**Fig. 8:**
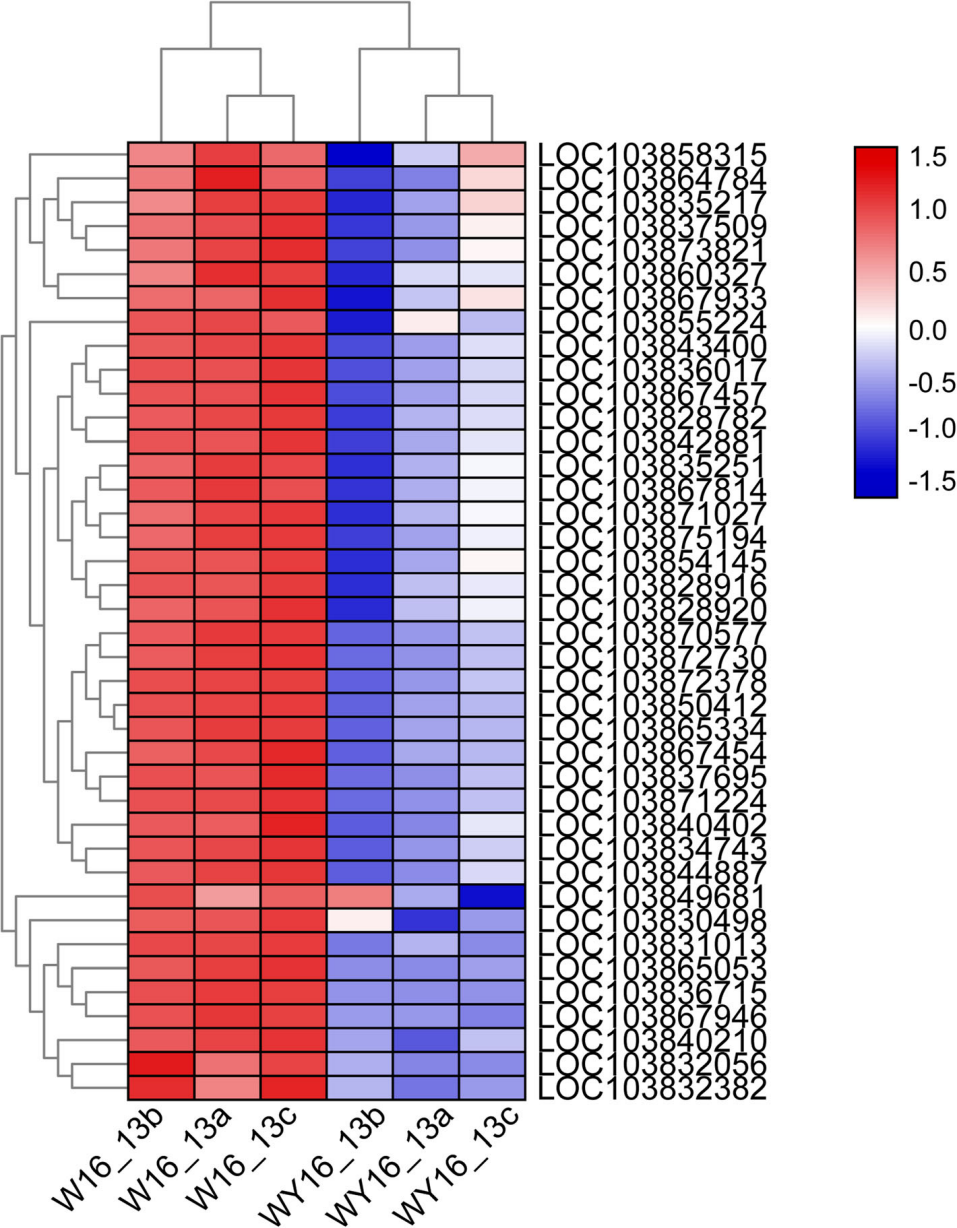
Heat map of the photosynthesis- and photosynthesis antenna protein-related DEGs in the leaves of W16_13a, WY16_13b, and WY16_13c and WY16_13a, WY16_13b, and WY16_13c. Expression ratios are based on log_2_ FPKM values. Red and blue represent high or low expression levels, respectively, than those shown in white

In the photosynthesis pathway (brp00195 and brp00196), a total of 40 DEGs encoding core proteins of Photosystem II (PSII), Photosystem I (PSI), the light-harvesting chlorophyll protein complex (LHC), and the photosynthetic electron transport were all downregulated in the WY16–13 mutant (Fig. 8). In PSII, the DEGs included oxygen-evolving enhancer protein 1 (Psb O), oxygen-evolving enhancer protein 2 (Psb P), Psb W protein (Psb W), psbY protein (Psb Y), and 10 kDa polypeptide (Psb R). In PSI, the DEGs included subunit II (Psa D), subunit IV A (Psa E), subunit III (Psa F), subunit V (Psa G), subunit VI (Psa H), and subunit Psa K, Psa O, and Psa N. In photosynthetic electron transport, the DEGs included ferredoxin (Pet F) and plastocyanin (Pet E). In addition, 15 genes encoding the chlorophyll a/b-binding protein were suppressed in the WY16–13 mutant (Additional file 5: Table S2). These results suggest that suppression of these photosynthetic genes was responsible for defects in chloroplast development in the WY16–13 mutant.

**Fig. 9:**
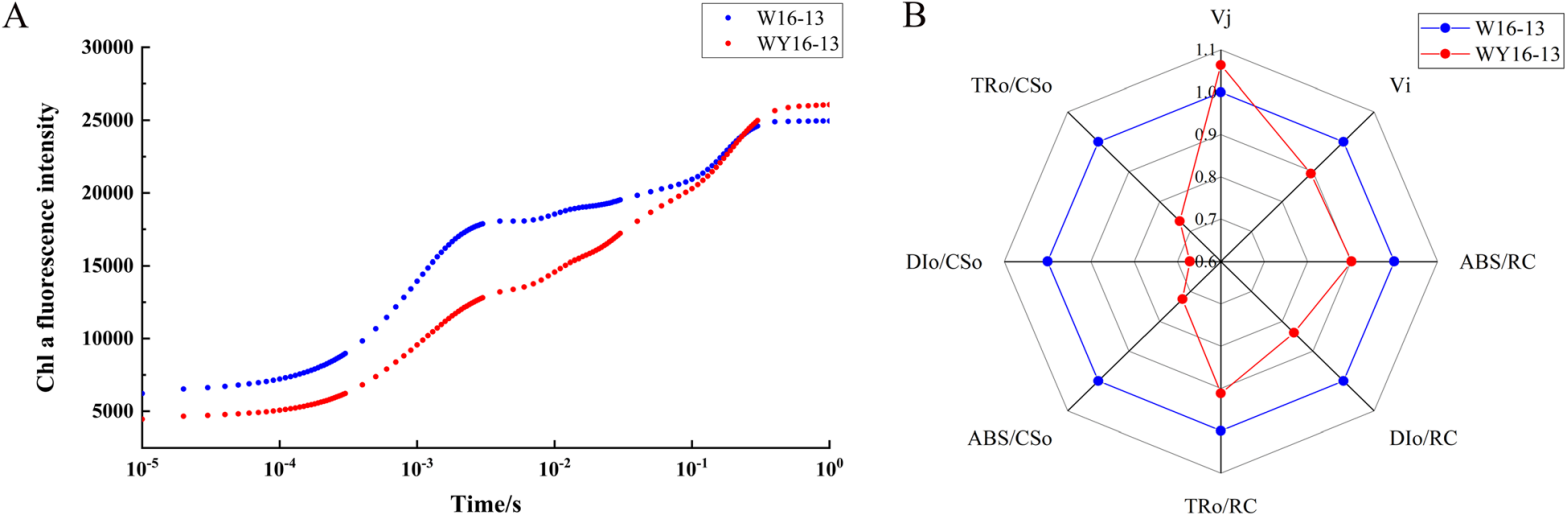
Fast Chl a fluorescence transient (OJIP) plotted on logarithmic time scale (0.00001–1 s) measured in WY16–13 and W16–13 (**a**), and Chl fluorescence kinetic parameters of WY16–13 and W16–13 (**b**)

The Chl a fluorescence transient reflects the effect of PSII after quantitatively analyzing changes in the OJIP curve (Fig. 9a). The chlorophyll fluorescence parameters of “WY16–13” and “W16–13” are shown in Fig. 9b and Table 3. The results show that the levels of F_0_, Fm, ABS/RC, and DI_o_/RC of WY16–13 were significantly lower than W16–13. Compared with W16–13, F_0_, F_m_, ABS/RC, and DI_o_/RC levels in WY16–13 decreased by 29.36, 21.88, 14.81, and 25.40%, respectively. Conversely, F_v_/F_m_, PI_abs_, and PI_total_ levels in WY16–13 were significantly higher than W16–13, which increased by 1.86, 37.12, and 154.36%, respectively.

**Table 3:** Chl fluorescence kinetic parameters of WY16–13 and W16–13

Chlorophyll fluorescence kinetic parameters	W16–13	WY16–13
F_0_	4359.42 ± 112.49^**^	3079.71 ± 337.95
F_m_	30,025.85 ± 845.46^**^	23,454.71 ± 2585.67
F_v_/F_m_	0.85 ± 0.001	0.87 ± 0.003^**^
ABS/RC	0.81 ± 0.04^**^	0.69 ± 0.03
DI_o_/RC	0.12 ± 0.006^**^	0.09 ± 0.006
V_j_	0.29 ± 0.02	0.31 ± 0.02
PI_abs_	16.38 ± 2.58	22.46 ± 4.01^**^
PI_total_	13.61 ± 2.74	34.62 ± 3.91^**^

Error bars indicate means ± SD based on three independent experiments. Significant differences were determined using the Student’s *t* test in WY16–13 compared with W16–13 (**p < 0.01)

### Quantitative real-time PCR (qRT-PCR) analysis

**Fig. 10:**
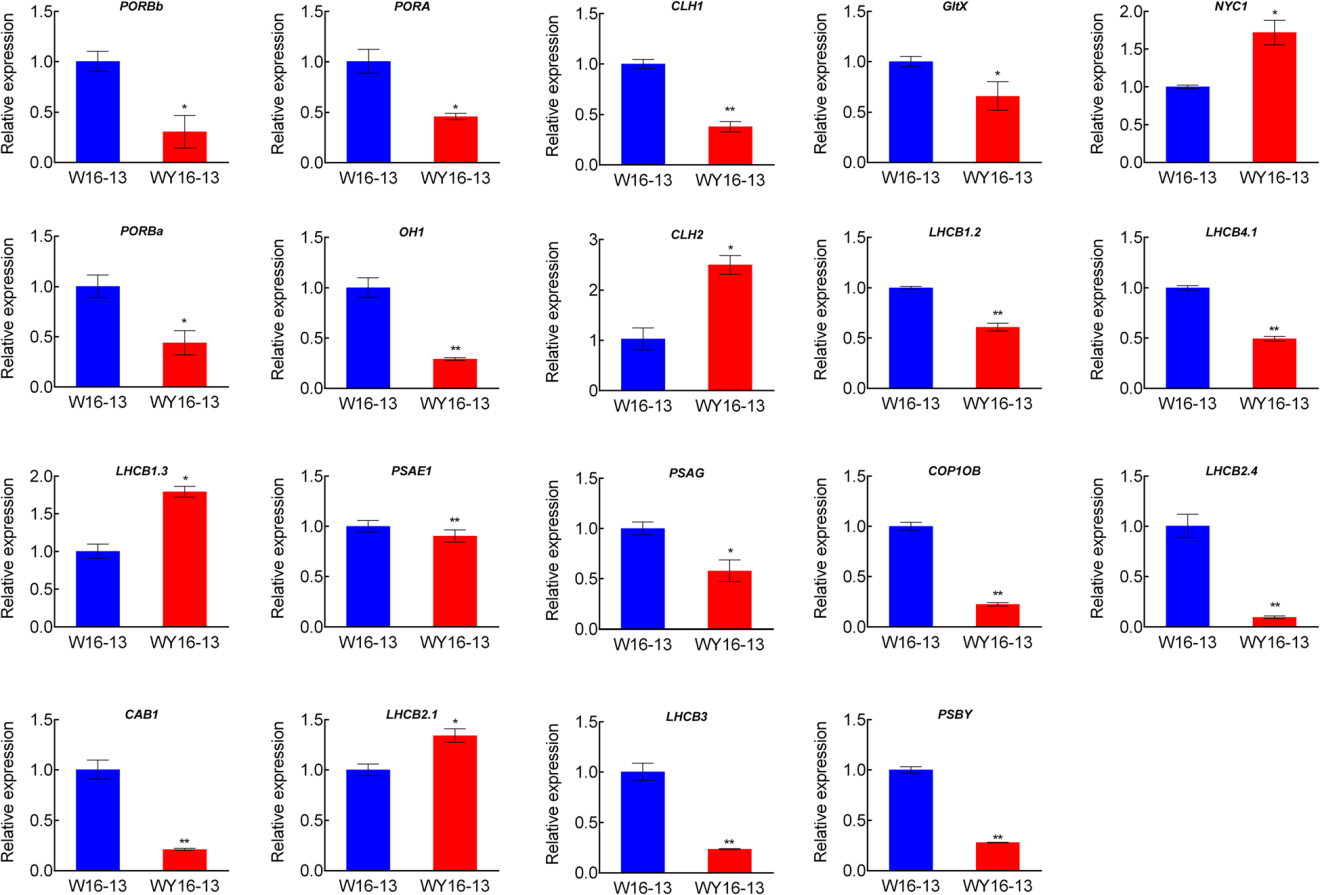
qRT-PCR of 19 DEGs in “WY16–13” and “W16–13” leaves. Error bars indicate means ± SD based on three independent experiments. Significant differences were determined using the Student’s *t* test in WY16–13 compared with W16–13 (^*^ *p* < 0.05, ^**^ *p* < 0.01)

To validate the reliability of the DEG expression, 20 DEGs were randomly selected for qRT-PCR analysis. The expression patterns revealed by qRT-PCR analysis were similar to those obtained by RNA-Seq for the same genes (Fig. 10). These findings indicate that the RNA-seq results of the present study are reliable for all kinds of analysis.

## Discussion

Chl deficiency and abnormal chloroplast development can lead to the plant leaf yellowing phenotype, which is closely related to photosynthesis. WY16–13 is a spontaneous yellow-green leaf color mutant of the cultivar W16–13 in wucai that presents a yellow-green leaf phenotype during the entire growth period. In this study, we conducted comprehensive biochemical analysis and transcriptome profiling of WY16–13 and W16–13 to obtain insights into leaf color variations at the transcriptional level and complex biological processes.

Leaves are the economically significant and utilized parts of vegetable plants and are also the primary sites of photosynthesis. Changes in leaf color are mainly determined by complex biological processes. Chl is the main pigment that harvests solar energy in leaf tissues. In this study, comparative analysis of photosynthetic pigment content in the leaves of WY16–13 and W16–13 revealed that the Chl and Car contents in WY16–13 were significantly lower than W16–13. We hypothesize that the yellow-green phenotype of WY16–13 leaves is caused by a deficiency in Chl, which has also been reported in *Arabidopsis* [6], soybean [22], rice [23], and cucumber [24].

Chl metabolism is determined by complex biological processes, and blockage of any step in this process can lead to a decrease in Chl content, which in turn results in a change in leaf color. Comparative transcriptome profiling of WY16–13 and W16–13 revealed that three genes encoding POR were downregulated (Fig. 7). POR is a key enzyme for Chl biosynthesis that catalyzes the photoreduction of protochlorophyllide to chlorophyllide. It has been reported that mutations in the *PORB* gene lead to a yellow/white leaf variegation phenotype in rice [5]. In addition, in *Arabidopsis*, the transcriptional activity of PORB and PORC in the yellow tissues of variegated mutants is either absent or very low [25]. These results suggest that inhibition of *POR* in the yellow leaf mutant reduces Chl content and leads to the yellow leaf phenotype. Moreover, two genes encoding CLH and one gene encoding NYC were upregulated in yellow leaf plants. Chlorophyllase is considered to be a rate-limiting enzyme that catalyzes the hydrolysis of chlorophyll to form chlorophyllide [26]. NYC1 protein is a chlorophyll b reductase that is highly similar to short-chain dehydrogenases/reductases [27]. Mutation of the *NYC1* gene leads to a stay-green phenotype during senescence in rice [28]. A previous study on *Cymbidium* orchids has suggested that the yellow-green leaf phenotype may be related to the upregulated expression of *CLH* [29]. Our results indicate that the upregulated expression of the *CLH* and *NYC1* genes in WY16–13 may accelerate Chl breakdown, lead to yellowing of plant leaves.

In addition, the *HO1* gene in WY16–13, which encodes heme oxygenase, was significantly (log_2_FoldChange = − 5.01) downregulated. Heme oxygenase is an important rate-limiting enzyme of heme metabolism and can cleave protoheme to form biliverdin, which in turn releases Fe^2+^ and carbon monoxide [30]. Disruption of heme metabolism in plants may inhibit Chl biosynthesis and cause leaf chlorosis [31]. In the rice yellowing mutant *ylc2*, a fragment deletion of the *osHO2* by map cloning resulted in the yellowing of young leaves [32]. By comparing and analyzing the content of Chl metabolism intermediate products, we found that the content of eight intermediate products in WY16–13 was significantly lower than W16–13 (Fig. 4). The Chl biosynthesis-hindered site of WY16–13 does not occur in the process from ALA to Chl. Therefore, the lack of Chl in WY16–13 may be caused by a disruption of heme metabolism. Excessive heme accumulation inhibits the activity of glutamyl-tRNA and the synthesis of ALA, thereby reducing the overall rate of tetrapyrrole biosynthesis, ultimately leading to a decrease in Chl synthesis. The rice *ygl2* mutant has an insertion mutation in the *heme oxygenase 1*, which leads to a significant reduction of *HO1* expression level, resulting in a yellow-green leaf phenotype [33]. Similar results have been found in *hy1* mutants of *A. thaliana* [34].

Chloroplasts are cytoplasmic organelles in eukaryotic cells that consist of a chloroplast membrane, thylakoid, and matrix and are the sites of photosynthesis [35]. Chloroplasts contain a highly folded thylakoid membrane system that includes the photosystems (PSII and PSI) and the cellular structure responsible for the generation of proton motility [36]. Metabolism and accumulation of Chl are inseparable from the normal development of chloroplasts. Leaf color yellowing may be related to the development of chloroplasts. Proteins plays an important role in the process of chloroplast development and differentiation [37]. Change in the coding region, synthesis, transportation, recognition, and binding of chloroplast proteins will directly or indirectly affect the synthesis and accumulation of Chl, which in turn results in leaf color variations [38]. Based on transcriptome data, we identified 40 DEGs that are related to photosynthesis between WY16–13 and W16–13. The DEGs are enriched in the photosynthetic pathway of plants such as (PSI, PSII, light harvesting complexes, cytochrome b6/f, and ATP synthase) and all downregulated (Fig. 8). These results are concordant to the findings of our previous analysis of the ultrastructure of chloroplasts, indicating that yellowing of leaves is largely affected by abnormal chloroplast development.

In higher plants, early light-induced proteins (ELIPs) are nuclear-encoded light stress-induced proteins in the thylakoid membrane system, where these are proposed to function in photoprotection [39–41]. The accumulation of ELIP transcripts and proteins is related to the degree of photoinactivation and photodamage of the PSII reaction center [42]. In WY16–13, the accumulation of *ELIP1* mRNA was 2.06-fold higher compared with W16–13, resulting in leaf color variations (Additional file 6: Table S3). Previous studies on *Arabidopsis* have suggested that the activity of glutamyl tRNA reductase, CHLH, and CHLI is reduced in *ELIP2* gene overexpression plants, resulting in a decrease in plant Chl content. Therefore, changes in the expression of genes related to carbon metabolism and photosynthesis in mutants may lead to abnormal chloroplast development and reduced Chl content. In addition, the Golden 2-like (GLK) families have been reported to be involved in the positive regulation of chloroplast development [43, 44]. In this study, we identified three GLK transcription factors, including two *GLK1* genes (*LOC103842594* and *LOC103828162*) and one *GLK2* gene (*LOC103827922*) (Additional file 6: Table S3). It is worth noting that the *GLK1* gene was upregulated 106.09-fold in the mutant. The *GLK* transcription factors encode transcriptional activators that promote the expression of nuclear-encoded photosynthetic genes that are required for Chl biosynthesis and light harvesting functions [45]. In *Arabidopsis*, the *glk1*-*glk2* double mutants exhibit a pale green phenotype and its chloroplasts lack thylakoid membranes and grana [46, 47]. These results further indicate that the expression levels of *GLKs* are closely related to chloroplast development and Chl biosynthesis.

## Conclusions

In summary, the physiological characteristics of the yellow-green leaf mutant “WY16–13” and normal green color cultivar “W16–13” were analyzed. The lower Chl contents and abnormal ultrastructure of chloroplasts in the leaves of WY16–13 suggested that Chl biosynthesis was partially inhibited. We performed transcriptome analysis by RNA-seq to elucidate the molecular mechanism underlying Chl metabolism and chloroplast development in the yellow-green leaf mutant. We identified nine DEGs that were related to the porphyrin and Chl metabolism. Among these, Chl biosynthesis genes, including *LOC103844881*, *LOC103861694*, and *LOC103867162* were downregulated in the mutant, while Chl degradation genes, including *LOC103848843*, *LOC103833353*, and *LOC103839225*, were upregulated. In addition, the downregulated *HO1* gene, which encodes heme oxygenase, caused heme accumulation and resulted in blockage of Chl synthesis. The results in this study provide molecular evidence for the development of the yellow-green leaf phenotype as well as insights into using the yellow leaf trait as marker for breeding.

## Methods

### Plant materials

The green leaf *Brassica campestris* L. cultivar (wild-type, W16–13) and the yellow-green leaf *B. campestris* L. cultivar (mutant, WY16–13) were used in the present study. The experiment was conducted in the breeding basin in Hefei, Anhui Province, China (north latitude 31.86, east longitude 117.26). Seedlings were planted in the growth chamber of 24 ± 1 °C (day) and 16 ± 1 °C (night) with a relative humidity of 75–80% and the light intensity was 300 μmol·m^− 2^·s^− 1^ in a 14-h/10-h light/dark photoperiod. After 20 days of transplanting, the third fully unfolded leaves from the center were collected for determination of photosynthetic efficiency and assessment of chloroplast ultrastructure. The samples were immediately frozen in liquid nitrogen after collection and stored at − 80 °C for physiological and biochemical experiments.

### Morphological observation

Morphological observation was performed every five days after planting. Leaf color values were estimated using a Chroma meter (CR-400-C, Konica Minolta Sensing Americas, Inc., Ramsey, NJ, USA) on the upper surface of the third unfolded leaf. A total of 10 plants were measured, and the two phenotypes were repeated thrice at the same leaf position. Plant height and expansion were measured with a rectilinear scale. Twenty WY16–13 and W16–13 plants with consistent growth and robust growth were measured as replicates.

### Measurement of Chl metabolite content

Chl was extracted with a mixture containing acetone, ethanol, and water (4.5:4.5:1, volume ratio) according to Strain et al., with minor modifications [48]. Fresh leaf (0.2 g) was extracted in the dark for 26 h, and the absorbance was determined by UV-vis spectrophotometer (TU1950, PERSEE, Beijing, China) at wavelengths of 665, 649, and 470 nm.

Glu content was estimated using a Solarbio reagent kit (Cat #BC1580, Beijing Solarbio Science & Technology Co., Ltd., China). The content of ALA was measured as described by Mauzerall et al. [49]. PBG, Urogen III, and Coprogen III contents were measured according to Bogorad [50]. Proto IX, Mg-Proto IX, and Pchlide contents were measured according to Hodgins et al. [51].

### Measurement of Chl a fluorescence transients

The fluorescence parameters were measured using a continuous excitation fluorometer Pocket Plant Efficiency Analyzer (PEA, Hansatech, UK). The initial fluorescence F_0_ and the maximal fluorescence F_m_, which respectively represented different fluorescence yields as the reaction center of PSII is fully opened or closed, were measured after the third functional leaves of the plants adapting in the dark for about 30 min. A fast Chl fluorescence induction curve was analyzed with Biolyzer 3.0 software (Bioenergetics Lab., Geneva, Switzerland).

### Transmission electron microscopy

Fresh functional leaf sections (≤ 2 mm × 5 mm) were fixed with 2.5% glutaraldehyde in 0.1 M PBS (sodium phosphate buffer, pH 7.0) for 12 h at 4 °C, and then washed thrice with 0.1 M PBS (pH 7.0). The samples were post fixed with osmium tetroxide (1%, w/v) for 2 h and again washed thrice by 0.1 M PBS (pH 7.0). Samples were dehydrated across an alcohol gradient (30, 50, 70, 80, 90, and 95%) for 15 min at each concentration, then treated with absolute ethanol for 20 min, and finally transferred to absolute acetone for 20 min. The samples were infiltrated and embedded in Spurr’s epoxy resin. After staining with uranyl acetate followed by lead citrate, the tissues were observed on a transmission electron microscope (HT-7700, Hitachi, Tokyo, Japan) at an accelerating voltage of 80.0 kV.

### RNA extraction, library construction, and RNA-seq

Total RNA was extracted from the W16–13 and WY16–13 leaf samples using a mirVana miRNA isolation kit (Ambion, TX, USA) according to the manufacturer’s instructions. The integrity of extracted RNA was assessed using an Agilent 2100 Bioanalyzer (Agilent Technologies, Santa Clara, CA, USA). Sequencing libraries were prepared using TruSeq Stranded mRNA LT Sample Prep Kit (Illumina, San Diego, CA, USA) following the manufacturer’s instructions. Then, these libraries were sequenced on an Illumina HiSeq™ 2500 platform (Biomarker Biotech, Beijing, China).

### Quality control and mapping of reads

The transcriptome sequencing and analysis were conducted by OE Biotech Co., Ltd. (Shanghai, China). The raw reads were processed to remove low-quality reads, adapters, and reads with poly-A or ploy-N to obtain clean reads using Trimmomatic (version 0.36) [52]. Then, clean reads were mapped to the *Brassica rapa* reference genome (http://brassicadb.org/brad/) using hisat2 (version 2.2.1.0) [53].

### Gene expression analysis

Gene expression level analysis was performed using DESeq (version 1.18.0) [54] R package, and genes were normalized with FPKM [55] using cufflinks (version 2.2.1) [56]. Genes with *p*-value < 0.05 and fold change > 2 or < 0.5 were considered DEGs between samples. Hierarchical cluster analysis of DEGs was performed to assess genes expression pattern. GO enrichment analysis of DEGs was performed using the GOseq R package. The enrichment of the DEGs in KEGG pathways was assessed using KOBAS software [57, 58].

### Validation of DEGs by qRT-PCR

Accuracy of transcriptome sequencing data was validated by qRT-PCR. Total RNA was extracted from leaves using a plant RNA extraction kit (Takara Biomedical Technology Co., Beijing, China). First-strand cDNA was synthesized using the PrimeScript™ RT reagent kit (TaKaRa). qRT-PCR was performed in a 20-μL reaction volume containing 1 μL of 100 ng cDNA, 1 μL each of 10 μM forward and reverse primers, 10 μL SYBR Premix Ex Taq II (Takara), and filled with 7 μL ddH_2_O. Gene-specific primers were designed using Primer Software version 5.0 (Premier Biosoft International, CA, USA). The primers used for qRT-PCR are listed in Additional file 7: Table S4. *BnaActin* gene was used as reference [21]. Relative expression levels were calculated as 2^-∆∆CT^ [59].

### Statistical analysis

Data were expressed as the mean ± SD with three biological replicates. All of the data was subjected to an analysis of variance (ANOVA). The mean separation was performed using the Student’s *t* test with a significance level of *p* < 0.05. Analyses were conducted using SPSS v22.0 for Windows (SPSS Inc., USA). The related figures were drawn using GraphPad Prism v6.0 (http://www.graphpad.com/scientific-software/prism/) and Origin Pro v9.1 software (OriginLab Corp., MA, USA).

## Supplementary Information

**Additional file 1: Figure S1**. Heat map of correlation coefficient between six samples.

**Additional file 2: Figure S2**. Principal component analysis of six samples.

**Additional file 3: Figure S3**. Comparison of the distribution of differentially expressed genes and all genes at KEGG Level 2. The Y-axis represents the Level 2 pathway term; The X-axis represents the ratio (%) of the total number of genes annotated to each Level 2 metabolic pathway (differentially expressed genes) and all genes annotated to the KEGG pathway.

**Additional file 4: Table S1**. DEGs of Porphyrin and chlorophyll metabolism.

**Additional file 5: Table S2**. DEGs of photosynthesis and photosynthesis–antenna proteins.

**Additional file 6: Table S3**. The expression patterns of ELIPs and GLKs.

**Additional file 7: Table S4**. Primer sequences for qRT-PCR.

## Data Availability

The dataset supporting the conclusions of this article is available in the NCBI’s BioProject database [PRJNA683756].

## Abbreviations

Chl: Chlorophyll
DEGs: Differentially expressed genes
GO: Gene Ontology
KEGG: Kyoto Encyclopedia of Genes and Genomes
Car: Carotenoid
Glu: Glutamate
ALA: 5-aminolevulinic acid
PBG: Porphobilinogen
Urogen III: Uroporphyrinogen III
Coprogen III: Coproporphyrinogen III
Proto IX: Protoporphyrin IX
Mg-Proto IX: Mg-protoporphyrin IX
Pchlide: Protochlorophyllide
qRT-PCR: Quantitative real-time polymerase chain reaction
LHC: Light-harvesting chlorophyll protein complex
PSII: Photosystem II
PSI: Photosystem I
