# Supplementary figures and images for: Characterization and transcriptomic analysis of a novel yellow-green leaf wucai (Brassica campestris L.) germplasm
Source: BMC Genomics. 2021 Apr 12;22:258. doi: 10.1186/s12864-021-07573-7 (PMC8040211; doi:10.1186/s12864-021-07573-7)

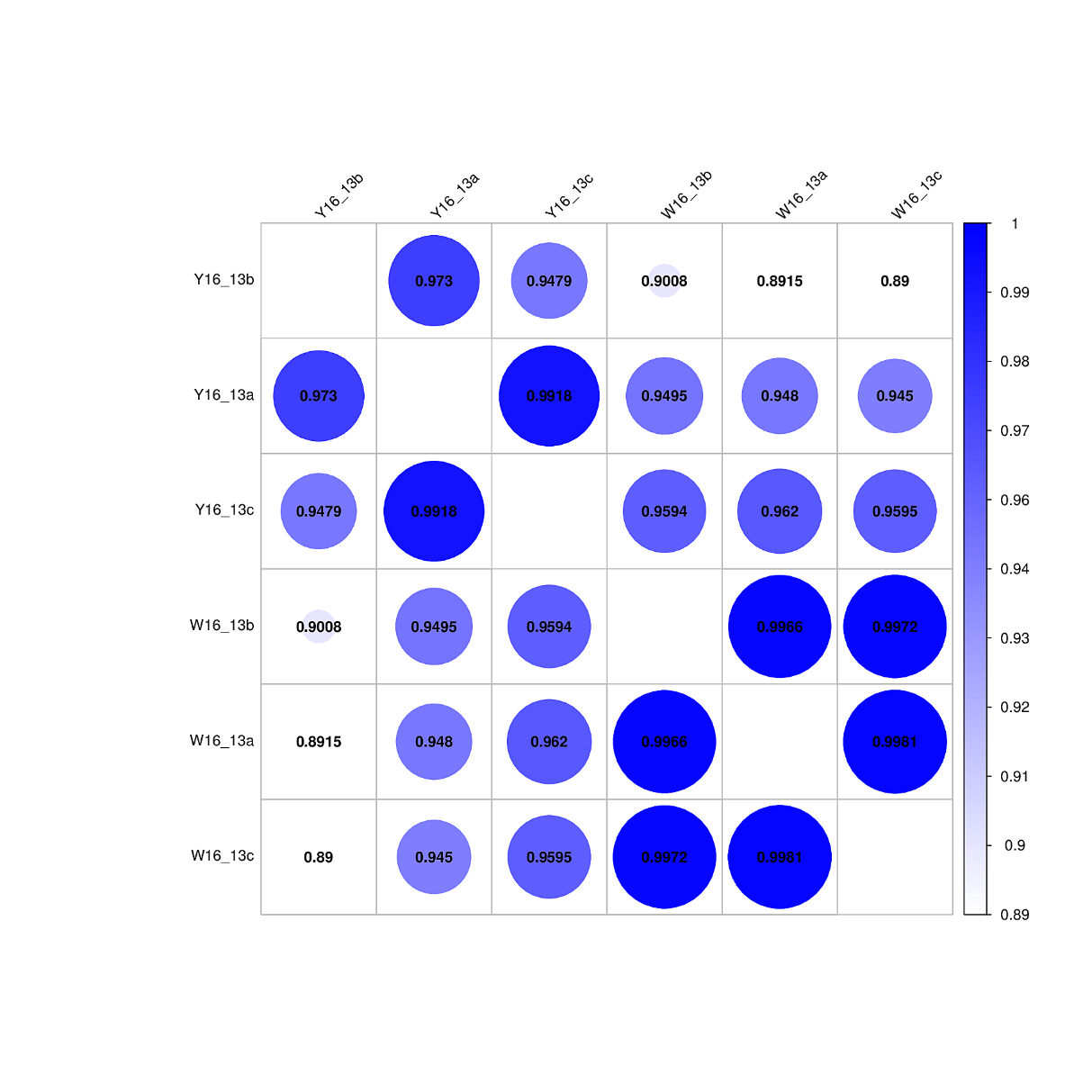

Supplement: Supplementary file 1 — Additional file 1: Figure S1. Heat map of correlation coefficient between six samples. [file 12864_2021_7573_MOESM1_ESM.docx]

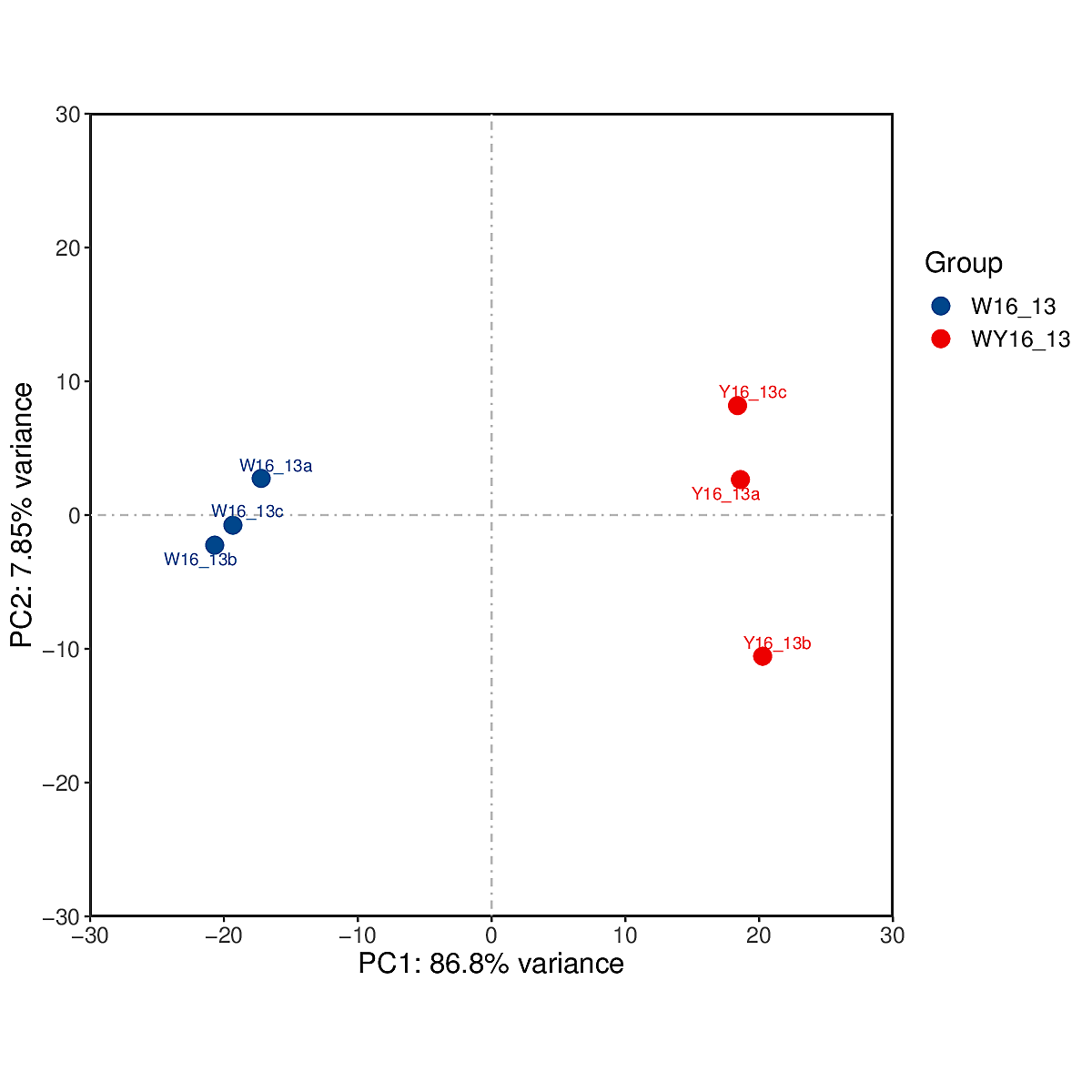

Supplement: Supplementary file 2 — Additional file 2: Figure S2. Principal component analysis of six samples. [file 12864_2021_7573_MOESM2_ESM.docx]

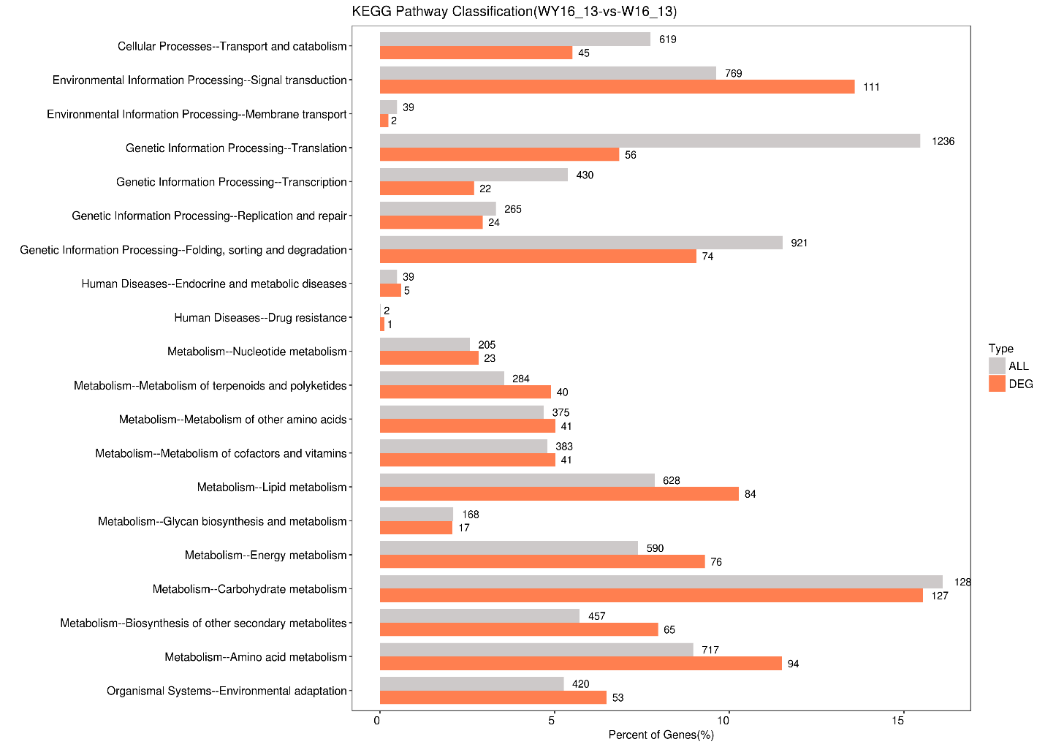

Supplement: Supplementary file 3 — Additional file 3: Figure S3. Comparison of the distribution of differentially expressed genes and all genes at KEGG Level 2. The Y-axis represents the Level 2 pathway term; The X-axis represents the ratio (%) of the total number of genes annotated to each Level 2 metabolic pathway (differentially expressed genes) and all genes annotated to the KEGG pathway. [file 12864_2021_7573_MOESM3_ESM.docx]
