# Supplementary material for: Characterization and transcriptomic analysis of a novel yellow-green leaf wucai (Brassica campestris L.) germplasm
Source: BMC Genomics. 2021 Apr 12;22:258. doi: 10.1186/s12864-021-07573-7 (PMC8040211; doi:10.1186/s12864-021-07573-7)
Supplement: Supplementary file 4 — Additional file 4: Table S1. DEGs of Porphyrin and chlorophyll metabolism. [file 12864_2021_7573_MOESM4_ESM.docx]

| Gene_ ID | *P* val | Up  Down | Description | Synonym |
| --- | --- | --- | --- | --- |
| LOC103848843 | 9.19E-06 | Up | chlorophyllase-2, chloroplastic | CLH2 |
| LOC103854720 | 4.75E-05 | Up | glutamate--tRNA ligase, cytoplasmic | GltX |
| LOC103833353 | 0.023049137 | Up | probable chlorophyll(ide) b reductase NYC1, chloroplastic | NYC1 |
| LOC103839225 | 0.005667918 | Up | chlorophyllase-2, chloroplastic | CLH2 |
| LOC103847911 | 1.18E-06 | Down | heme oxygenase 1, chloroplastic | HO1 |
| LOC103872768 | 6.48E-10 | Down | chlorophyllase-1 | CLH1 |
| LOC103844881 | 0.015429744 | Down | protochlorophyllide reductase A, chloroplastic | PORA |
| LOC103861694 | 4.63E-09 | Down | protochlorophyllide reductase B, chloroplastic-like | PORB |
| LOC103867162 | 1.31E-09 | Down | protochlorophyllide reductase B, chloroplastic | PORB |
