# Supplementary material for: Characterization and transcriptomic analysis of a novel yellow-green leaf wucai (Brassica campestris L.) germplasm
Source: BMC Genomics. 2021 Apr 12;22:258. doi: 10.1186/s12864-021-07573-7 (PMC8040211; doi:10.1186/s12864-021-07573-7)
Supplement: Supplementary file 5 — Additional file 5: Table S2. DEGs of photosynthesis and photosynthesis–antenna proteins. [file 12864_2021_7573_MOESM5_ESM.docx]

| Gene_ ID | *P* val | Up  Down | Description | Synonym |
| --- | --- | --- | --- | --- |
| Photosynthesis - antenna proteins |  |  |  |  |
|  |  |  |  |  |
| LOC103828916 | 6.53E-131 | Down | Chlorophyll a-b binding protein 1, chloroplastic | LHCB1.3 |
| LOC103828920 | 3.94E-17 | Down | Chlorophyll a-b binding protein 1, chloroplastic | LHCB1.3 |
| LOC103829997 | 1.37E-28 | Down | Chlorophyll a-b binding protein 6, chloroplastic | LHCA1 |
| LOC103835251 | 1.34E-37 | Down | Chlorophyll a-b binding protein 3, chloroplastic | LHCB1.2 |
| LOC103836017 | 1.82E-28 | Down | Chlorophyll a-b binding protein CP24 10A, chloroplastic | CAP10A |
| LOC103837002 | 8.39E-89 | Down | Chlorophyll a-b binding protein 2.4, chloroplastic | LHCB2.4 |
| LOC103838443 | 3.19E-69 | Down | Photosystem I chlorophyll a/b-binding protein 3-1, chloroplastic | LHCA3 |
| LOC103841392 | 4.10E-31 | Down | Chlorophyll a-b binding protein 6, chloroplastic | LHCA1 |
| LOC103842881 | 2.08E-64 | Down | Chlorophyll a-b binding protein CP24 10A, chloroplastic | CAP10A |
| LOC103844887 | 2.78E-60 | Down | Chlorophyll a-b binding protein 3, chloroplastic | LHCB3 |
| LOC103850412 | 4.74E-67 | Down | Chlorophyll a-b binding protein CP29.1, chloroplastic | LHCB4.1 |
| LOC103857533 | 2.74E-46 | Down | Chlorophyll a-b binding protein 1, chloroplastic | CAB1 |
| LOC103858561 | 4.86E-40 | Down | Chlorophyll a-b binding protein CP26, chloroplastic | LHCB5 |
| LOC103860327 | 0.0059129 | Down | Chlorophyll a-b binding protein 2.1, chloroplastic | LHCB2.1 |
| LOC103862822 | 3.05E-59 | Down | Photosystem I chlorophyll a/b-binding protein 2, chloroplastic | LHCA2 |
| LOC103865334 | 7.71E-64 | Down | Chlorophyll a-b binding protein 1, chloroplastic | CAB1 |
| LOC103867454 | 3.54E-94 | Down | Chlorophyll a-b binding protein 1, chloroplastic | CAB1 |
| LOC103867457 | 1.59E-18 | Down | Chlorophyll a-b binding protein 1, chloroplastic | CAB1 |
| LOC103867814 | 9.14E-30 | Down | Chlorophyll a-b binding protein 1, chloroplastic | CAB1 |
| LOC103870577 | 2.69E-80 | Down | Chlorophyll a-b binding protein CP29.2, chloroplastic | LHCB4.2 |
| LOC103872378 | 8.56E-31 | Down | Chlorophyll a-b binding protein CP24 10A, chloroplastic | CAP10A |
| LOC103873241 | 1.22E-89 | Down | Chlorophyll a-b binding protein 4, chloroplastic | LHCA4 |
| LOC103875194 | 2.57E-127 | Down | Chlorophyll a-b binding protein 2.4, chloroplastic | LHCB2.4 |
| Photosynthesis |  |  |  |  |
|  |  |  |  |  |
| LOC103828782 | 3.286E-47 | Down | Photosystem I reaction center subunit III, chloroplastic | PSAF |
| LOC103829589 | 1.567E-41 | Down | Oxygen-evolving enhancer protein 1-1, chloroplastic | PSBO1 |
| LOC103832382 | 4.557E-09 | Down | Photosystem II 10 kDa polypeptide, chloroplastic | PSBR |
| LOC103832805 | 0.0009125 | Down | Photosystem I reaction center subunit VI, chloroplastic | PSAH |
| LOC103836470 | 1.94E-26 | Down | Oxygen-evolving enhancer protein 2, chloroplastic | PSBP |
| LOC103836610 | 3.64E-14 | Down | Photosystem II repair protein PSB27-H1, chloroplastic | PSB27-1 |
| LOC103836715 | 2.889E-15 | Down | Photosystem I reaction center subunit II-1, chloroplastic | psaD1 |
| LOC103837509 | 5.067E-28 | Down | Photosystem I reaction center subunit N, chloroplastic | PSAN |
| LOC103837695 | 6.545E-19 | Down | Oxygen-evolving enhancer protein 1-1, chloroplastic | PSBO1 |
| LOC103839762 | 2.053E-45 | Down | Oxygen-evolving enhancer protein 3-2, chloroplastic | PSBQ2 |
| LOC103843400 | 9.395E-30 | Down | Photosystem I subunit O | PSAO |
| LOC103843499 | 5.38E-13 | Down | Oxygen-evolving enhancer protein 2, chloroplastic | PSBP |
| LOC103844017 | 1.518E-36 | Down | Oxygen-evolving enhancer protein 2, chloroplastic | PSBP |
| LOC103844493 | 4.194E-27 | Down | Photosystem II repair protein PSB27-H1, chloroplastic | PSB27-1 |
| LOC103855224 | 2.312E-05 | Down | Photosystem I reaction center subunit N, chloroplastic | PSAN |
| LOC103855346 | 2.357E-34 | Down | Oxygen-evolving enhancer protein 1-1, chloroplastic | PSBO1 |
| LOC103867933 | 4.122E-05 | Down | Oxygen-evolving enhancer protein 2-1, chloroplastic | PSBP1 |
| LOC103871027 | 8.415E-41 | Down | Photosystem I reaction center subunit V, chloroplastic | PSAG |
| LOC103833816 | 5.413E-10 | Down | Photosystem I reaction center subunit III, chloroplastic | PSAF |
| LOC103835217 | 4.345E-37 | Down | Photosystem I reaction center subunit psaK, chloroplastic | PSAK |
| LOC103871224 | 1.258E-21 | Down | Photosystem I reaction center subunit VI, chloroplastic | PSAH |
| LOC103872730 | 1.868E-28 | Down | Oxygen-evolving enhancer protein 1-2, chloroplastic | PSBO2 |
| LOC103873821 | 4.10E-23 | Down | Photosystem I reaction center subunit N, chloroplastic | PSAN |
