# Supplementary material for: Characterization and transcriptomic analysis of a novel yellow-green leaf wucai (Brassica campestris L.) germplasm
Source: BMC Genomics. 2021 Apr 12;22:258. doi: 10.1186/s12864-021-07573-7 (PMC8040211; doi:10.1186/s12864-021-07573-7)
Supplement: Supplementary file 6 — Additional file 6: Table S3. The expression patterns of ELIPs and GLKs. [file 12864_2021_7573_MOESM6_ESM.docx]

| Gene_ ID | *P* val | Fold Change | Up  Down | Description | Synonym |
| --- | --- | --- | --- | --- | --- |
| LOC103868967 | 2.31E-05 | 2.067757892 | Up | Early light-induced protein 1, chloroplastic | ELIP1 |
| LOC103842594 | 1.48E-04 | 0.378326295 | Down | Transcription activator GLK1-like | GLK1 |
| LOC103828162 | 1.50E-14 | 106.0942967 | Up | Transcription activator GLK1 | GLK1 |
| LOC103827922 | 4.65E-08 | 0.333549755 | Down | Transcription activator GLK2-like | GLK2 |
