# Supplementary material for: Characterization and transcriptomic analysis of a novel yellow-green leaf wucai (Brassica campestris L.) germplasm
Source: BMC Genomics. 2021 Apr 12;22:258. doi: 10.1186/s12864-021-07573-7 (PMC8040211; doi:10.1186/s12864-021-07573-7)
Supplement: Supplementary file 7 — Additional file 7: Table S4. Primer sequences for qRT-PCR. [file 12864_2021_7573_MOESM7_ESM.docx]

| Table S4  Primer sequences for qRT-PCR.   \| Gene name \| Primer name \| Primer sequence (5’->3’) \| \| --- \| --- \| --- \| \| *BnaActin* \| primer F \| TGGGTTTGCTGGTGACGAT \| \|  \| primer R \| TGCCTAGGACGACCAACAATACT \| \| LOC103867162 \| primer F \| TACACTCTTCCCTCCGTTT \| \|  \| primer R \| TCCCACACTTTACGAGCCTT \| \| LOC103844881 \| primer F \| GAGCTTCCCGACGAACAATC \| \|  \| primer R \| AGAACCACAAGCGAGAGAC \| \| LOC103872768 \| primer F \| GGAACTTACCCCGTCGTCT \| \|  \| primer R \| ATCACTTTTCCAGCATCGTC \| \| LOC103854720 \| primer F \| TGACCCTGTTTACTACCGTTG \| \|  \| primer R \| AACTCTCCTCAACCCCATA \| \| LOC103833353 \| primer F \| GTCATCGTTACATCTCGCAG \| \|  \| primer R \| GTCATCGTTACATCTCGCAG \| \| LOC103861694 \| primer F \| TTCAAAGCGTCTCATTATCGTC \| \|  \| primer R \| CAGTTTCTTCATGGTAACG \| \| LOC103847911 \| primer F \| ACAACATCTACTTCGCCCACA \| \|  \| primer R \| ATTCTTTTCTTCTCTCGTCCACT \| \| LOC103848843 \| primer F \| GCTGCTACAAAACGACGCCAT \| \|  \| primer R \| AAGCCATAGGAAGAGACATGCAAC \| \| LOC103835251 \| primer F \| CTGCGTCTTCCCTGAGTTGT \| \|  \| primer R \| TGAGTAGCCCAAATAGCC \| \| LOC103850412 \| primer F \| CCCTTCTCCATCTCGACG \| \|  \| primer R \| AGCCATCTTCTCAGGGTCA \| \| LOC103828920 \| primer F \| TTTGGCTATTTGGGCTACTC \| \|  \| primer R \| ACATAGCCAATCTTCCGTTC \| \| LOC103854145 \| primer F \| AACTCCGTGTCTTTCTTACC \| \|  \| primer R \| AGCCACAACTGATCCAACG \| \| LOC103871027 \| primer F \| GCCCATCCGTCGTCATAAGCC \| \|  \| primer R \| GTGACCAATAGAACCCCAAGC \| \| LOC103872378 \| primer F \| CCATCGCACCTTTCTCTTTC \| \|  \| primer R \| CGAAATTCTCAGCGGTCCT \| \| LOC103875194 \| primer F \| CAATCCAGCACTCCTCTTTCGC \| \|  \| primer R \| CTGTGTCCCAACCGTAGTCTCC \| \| LOC103867457 \| primer F \| TCCTCCCCTGCCTTAGCC \| \|  \| primer R \| TGTCCCATCCGTAGTCACC \| \| LOC103860327 \| primer F \| CCCTCAAGCCCTCCAACGA \| \|  \| primer R \| GTGTCCCAGCCGTAGTCTCC \| \| LOC103844887 \| primer F \| AACCCCGTCTTACCTCACC \| \|  \| primer R \| AAGATTTGTGAACCGGCTTT \| \| LOC103831013 \| primer F \| ACCACTAAAACCCACCGTCT \| \|  \| primer R \| TGTTGAAGAGCACCCATCC \| |
| --- | --- | --- | --- | --- | --- | --- | --- | --- | --- | --- | --- | --- | --- | --- | --- | --- | --- | --- | --- | --- | --- | --- | --- | --- | --- | --- | --- | --- | --- | --- | --- | --- | --- | --- | --- | --- | --- | --- | --- | --- | --- | --- | --- | --- | --- | --- | --- | --- | --- | --- | --- | --- | --- | --- | --- | --- | --- | --- | --- | --- | --- | --- | --- | --- | --- | --- | --- | --- | --- | --- | --- | --- | --- | --- | --- | --- | --- | --- | --- | --- | --- | --- | --- | --- | --- | --- | --- | --- | --- | --- | --- | --- | --- | --- | --- | --- | --- | --- | --- | --- | --- | --- | --- | --- | --- | --- | --- | --- | --- | --- | --- | --- | --- | --- | --- | --- | --- | --- | --- | --- | --- | --- | --- |
